# From inflammation to cancer

**DOI:** 10.1007/s11845-016-1464-0

**Published:** 2016-05-07

**Authors:** A. Korniluk, O. Koper, H. Kemona, V. Dymicka-Piekarska

**Affiliations:** Department of Clinical Laboratory Diagnostics, Medical University of Bialystok, Ul. Waszyngtona 15A, 15-276 Bialystok, Poland

**Keywords:** Acute inflammation, Chronic inflammation, Cancer, Adhesion molecules

## Abstract

**Background:**

The participation of inflammation in the progression of cancer for many years have been the subject of research.

**Methods:**

In the 19th century, there was evidence that an acute inflammation may inhibit the development of cancer. However, chronic inflammation affects the progression of the disease.

**Results:**

Today, it is known that inflammation and cancer use similar mechanisms of development such as severe cell proliferation or angiogenesis. It has been shown that prolonged presence of inflammatory cells and factors in the tumor microenvironment can accelerate its growth and inhibit apoptosis of transformed cells.

**Conclusion:**

In this article we present a brief history of the discovery mechanisms and potential links between acute and chronic inflammation and cancer.

## Introduction

In 1863, Rudolf Virchow put forward a hypothesis that infiltrated immune cells reflect the place where cancer lesions appear in the inflamed tissue [1]. Not earlier than a century later, Dvorak showed that carcinogenesis and inflammatory conditions have common developmental pathways, such as proliferation, increased cell survival and migration, and enhanced angiogenesis which are controlled by growth factors, proinflammatory cytokines and proangiogenic factors. Moreover, the author observed that the cells involved in inflammation also infiltrated cancer tissue [2, 3] and defined cancer as a “wound that does not heal”. Therefore, we believe that in order to fully elucidate the role of an inflammatory condition in carcinogenesis, first we should have a profound understanding of what inflammation really is.

## Short brief history

The discovery and understanding of inflammatory mechanisms dates back to the ancient times of Egyptians and Greeks. The first description of inflammation appears in the works of Aulus Cornelius Celsus, who defined four basic inflammatory signs and symptoms: redness, swelling, warmth and pain. In 2nd century AD, a Roman surgeon Galen defined the inflammatory state as a favorable early component of healing processes in injured tissues, leading to recovery. Some authors suggest that he introduced the fifth sign of inflammation, i.e., function loss. However, according to most scientists, the term *functio laesa* was introduced in 1871 by Virchow [4].

Although ancient scientists based their concepts mainly on observations and intuition and not on profound investigations, they initiated later experiments aimed to discover the mechanisms involved in the inflammatory process. The invention of a microscope was a breakthrough in their understanding, making it possible to observe the microcirculatory changes in inflamed tissues [5]. For instance, blood clotting “tendency” was discovered (Gaubius 1794) and angiogenesis was first described as the process of new vessel formation in healing tissue. In 1824, Dutrochet showed the ability of leukocytes to migrate within the blood and 15 years later Weyner described the process of leukocyte rolling on the endothelium. Molecular changes in the vascular walls were found to underlie the adhesion of leukocytes to the endothelium. In nineteenth century, Virchov propagated research into an inflammatory state [6]. Contrary to Galen, he described the inflammatory condition as a pathological process associated with the excessive inflow and proliferation of cells caused by the release of nutrients from damaged blood vessels. He was the first to observe the cellular nature of an inflammatory response. He also emphasized the involvement of leukocytes in inflammation, although he erroneously concluded that these cells were responsible for infection and tissue damage. A Virchov’s student, Cohnheim (1867) suggested that the increased “porosity” of blood vessels is associated with fluid escape to the extravascular space. Using a microscope he was the first to describe the sequences that occur during an inflammatory condition; however, like his master, he wrongly defined the functions of leukocytes [7]. It was only a Russian scientist, Miecznikow, who refuted these theories and proved that it was the invasion of a foreign agent that caused the migration of host cells to the infected site, phagocytosis and damage of pathogens. In the subsequent years, the role of inflammatory cells (including phagocytes) was described, whereas research conducted by Rocha e Silva showed the involvement of “chemical mediators” in the development of an inflammatory condition, which supplemented the knowledge of an inflammatory response [4].

Currently, we know that irrespective of the triggering agent, tissue damage activates signals that initiate and support the immune response of the host, able to eliminate a damaging agent and to repair tissues [8]. Pathogen invasion activates inflammatory cells, macrophages and mast cells that generate and release cytokines (TNF-α, IL-1β, IL-6) and proinflammatory chemokines (IL-8, MCP-1, MIP-1α), which together with chemoattractants (bacterial toxins) stimulate attraction of circulating leukocytes to the site of inflammation [9].

## Inflammatory condition step by step

The initial stage of an inflammatory response involves leukocyte marginalization. It takes place in small-diameter vessels, in which inflammatory cells do not migrate to the center but are pushed towards the vascular walls, where the process of their endothelial rolling begins. TNF-α, histamine and other mediators of inflammation stimulate translocation of P-selectin from Weibel–Palade corpuscles, which is later incorporated in the endothelial cell membrane. Selectin interacts with sialylated or fucosylated carbohydrate residues of the Lewis group antigens, which act as their ligands. PSGL-1 is best known among them [10]. However, any adequately modified glycoprotein can be a potential selectin receptor, like CD34, which having been transformed on the endothelial surface becomes L-selectin receptor on leukocytes [11]. Ligand binding to selectin is a dynamic process and the bonds being formed undergo rapid dissociation. Many times the binding of leukocytes to endothelium slows down their vascular flow from 1000 to 30 µm/s. A few hours after activation, E-selectin appears on the endothelial surface [12]. P-selectin mediates binding at *high shear stress*, whereas E-selectin takes part in slow leukocyte rolling (*low shear stress*). This is associated with partial leukocyte integrin activation [13]. The rolling leukocytes may have a direct contact with endothelium, due to which they may be activated by inflammatory factors (chemokines) present on the endothelial surface.

Chemokines are produced by endothelial cells or interstitial inflammatory cells and then transported to the vascular lumen, where they bind to heparin sulfate glycosaminoglycans on the endothelial surface. Contact of these proteins with specific leukocyte receptors (a subgroup of receptors conjugated with G protein) causes the transmission of a signal that activates integrins present on the inflammatory cells [14].

Integrins belong to a family of heterodimeric adhesion receptors that remain in the resting inactive conformation. Most integrins are the extracellular matrix proteins. During activation changes take place in the arrangement of these proteins, which allows binding to a ligand [15]. Leukocytes have on their surface members of the β2 family (CD18), whose main ligands, i.e., the immunoglobulin-like molecules, ICAM-1 and ICAM-2, are present on the endothelium [16]. The surface of lymphocytes and monocytes exhibits the β1 family integrins (VLA-4, CD49d/CD29, α4β1), which bind to VCAM-1 molecules [17]. Following activation, integrins bind leukocytes tightly to the endothelial surface, causing their complete arrest. Next, leukocytes “crawl” to the contact point of endothelial cells where they prepare for extravasation. The process of “crawling” is mediated by proteins belonging to the leukocyte and endothelial integrins, i.e., Mac-1 (CD11b/CD18) that bind ICAM-1. In the case of monocytes, the transmigration is mediated also by LFA-1 (CD11a/CD18) and ICAM-2 [18]. During diapedesis leukocytes assume the amoeboid shape as a result of cytoskeletal alterations and push their way between endothelial cells [19]. The stages of rolling, activation, adhesion and “crawling” are reversible and require heterophilic bonds. On the contrary, diapedesis is a no-return path, during which leukocytes interact due to homophilic bonds [20].

PECAM-1 is the first molecule reported to take part in transmigration (TEM). It shows a specific distribution on the cell surface. PECAM-1 on leukocytes has been found dispersed throughout the cell membrane, whereas on endothelial cells its highest level has been shown at the cell contact point. Apart from PECAM-1, such distribution is also characteristic of CD99 protein [21, 22]. Leukocytes’ migration to the extravascular space is associated with the fact that they have to pass through the interstitial tissue. It has been suggested that neutrophils and monocytes migrate through the basement membrane to the sites with the lowest expression of collagen IV and laminin 10. This process is to delimit injuries caused by proteolytic enzymes used by leukocytes during their passage to the site of damage. The process occurs when the inflammatory cell CD11a binds to pericyte-ICAM-1 to allow leukocytes to reach the sites with the lowest density of the collagen network [23]. Having reached the site of damage, leukocytes eliminate the pathogen through the activation of aerobic and anaerobic killing. If the process runs properly, the elimination of the inflammatory factor suppresses the inflammation thanks to the action of anti-inflammatory mediators. For instance, the production of proinflammatory prostaglandins is decreased and the generation of anti-inflammatory lipoxins that attract monocytes, IL-10 or TGF, is increased [24].

Neutrophils which appear early at the site of damage constitute the first line of body defense. To a large extent, they are able to eliminate a pathogen on their own. Other cells include monocytes and lymphocytes initiating healing and generation of immunological memory [25].

When the inflammatory cells are not able to eliminate a pathogen, acute inflammatory condition may turn chronic. This chronic phase is characterized by hyper-intensive leukocyte infiltration of injured tissues, which results in the formation of leukocyte infiltrates. They were first described by Virchov when he examined tumor tissues. He observed that the “lymphoreticular infiltrate reflected the origin of cancer at sites of chronic inflammation” [1]. The elucidation of the mechanisms responsible for the development of inflammation initiated the research into mutual relationship to neoplastic disease [26].

## Acute inflammatory condition: not always bad

The fact that inflammation can have a double role in the development of cancer was already observed in the 19th century (1868) by a German physician Burns. He described cases of severe streptococcal infection during which patients experienced regression of neoplastic disease. More than 20 years later, this fact was taken into consideration by Coley who created the so called “Coley’s toxin”, a suspension of gram-negative bacterial cells which he successfully administered to cancer patients. The pathogen-induced body stimulation results in the activation of the immune system, which not only fights the bacteria but also inhibits tumor growth. The role of TNF-α (tumor necrosis factor), whose release is stimulated, among others, by a bacterial toxin, LPS (lipopolysaccharide), has been proved [27]. Currently, induced acute inflammation is used in the therapy of squamous cancer of the bladder. Patients receive a special vaccine against tuberculosis containing an attenuated Mycobacterium bovis strain (Mycobacterium bovis bacillus Calmette–Guerin—BCG) directly to the urinary bladder [28]. Induced immune reaction is associated with cytokine secretion, including interleukin-1 (IL-1), IL-2, IL-5, IL-6, IL-8, IL-10, IL-12, IL-15, IL-18, TNF-α, GM-CSF and interferon (IFN)-γ [28]. Studies are being conducted on the potential use of Salmonella typhimurium containing vaccines in oncological therapy. Acute inflammatory condition is considered a physiological process and a body’s defensive response. Sometimes referred to as “therapeutic inflammation” can show anticancer effects. However, it should be remembered that pathogens try to avoid the defense systems of the host, which may lead to a chronic condition that often accompanies the neoplastic disease.

## Chronic inflammatory condition: always bad

“Pathological inflammation”, i.e., acute phase response of moderate intensity (chronic inflammation) is involved in neoplastic transformation and stimulation of cancer growth [29]. Already in 1828, Jean Nicholas Marjolin, a French surgeon, provided evidence for the involvement of chronic inflammation in the development of cancer. He observed the growth of squamous cancer around the open chronically inflamed wound. It is currently estimated that approximately 25 % of cancers are associated with chronic inflammation caused by infection or physicochemical agents [30]. Persistent gastritis induced by Helicobacter pylori increases the risk of stomach cancer even by 75 % [31], whereas types B and C hepatitis promote the formation of hepatocellular carcinoma [29]. Also noninfectious diseases may increase the risk of cancer. Cancers of the pancreas and prostate often follow chronic inflammation in these organs [32]. The closest relationship between chronic inflammation and cancer can be observed in chronic ulcerating colitis and Crohn’s disease, which increase the risk of colorectal cancer even tenfold [33]. Moreover, inflammation-based tumors are characterized by the presence of cells and inflammatory mediators, thus indicating that inflammation and cancer can be combined by the extrinsic and intrinsic pathways. In the former, cancer growth is associated with an ongoing inflammatory state. The latter is stimulated by genetic changes leading to the activation of oncogenes and inactivation of suppressor genes. Cells with an altered phenotype produce inflammatory mediators thus leading to the activation of immune response and development of inflammation [34].

In both models, carcinogenesis is a two-stage process. Cancer development is initiated when the DNA sequence in somatic cells undergoes genetic mutations that can be present in healthy tissues and remain occult for many years until another stimulus pushes the cells into the “promotion” stage [30]. This process may occur when the transformed cells are exposed to chemical irritants, such as phorbol esters, substances released at the site of injury, partial resection of the organ, hormones or chronic irritation and inflammation. Functionally, many “promoters” may directly or indirectly induce cell proliferation, recruitment of inflammatory cells, may increase production of reactive oxygen species, leading to oxidative DNA damage and decreasing its repair [35].

In the process of initiation normal cells undergo mutation. Usually a single change in DNA is insufficient, but 4–5 mutations can direct cells onto the neoplastic transformation pathway. Activated leukocytes release reactive oxygen species (ROS) and reactive nitrogen intermediates (RNI), which can induce DNA damage and genome instability [8]. The major role of inflammation in mutagenesis can be exemplified by the development of colorectal adenocarcinoma induced by inflammation and the action of an irritant, dextran sodium sulfate (DSS) [35]. DSS itself shows a weak carcinogenic effect, although the inflammatory state highly augments the effect [35]. Moreover, the inflammatory cells producing cytokines may induce overexpression of cytidine deaminase (activation-induced cytidine deaminase-AID). AID causes genome instability and increases the probability of mutations in the course of abnormal binding of double stranded DNA, i.e., when most frequently mutations form in oncogenesis-critical genes, such as TP53, c-myc and Bcl-6. AID involvement has been demonstrated in the formation of lymphomas, and gastric and liver cancers [36]. Damage to DNA can also lead to the inflammatory process and in consequence to cancer formation. The examples include DEN-induced hepatocellular carcinoma induced by DEN (diethylnitrosamine), which causes DNA damage resulting in necrotic cell death and inflammatory response that promotes cancer progression [37]. Many oncoproteins (RAS, Myc, RET) can also activate the signaling pathways which enhance the production of inflammatory cytokines and chemokines (IL-6, IL-8, IL-1β, CCL2, CCL20) [34]. The initiation stage lasts until an additional factor begins the stage of promotion, and thus growth from single cells to a fully developed primary cancer. The initial growth of cancer depends on the increased cell proliferation and limited apoptosis, and these two processes are stimulated by mechanisms closely related to inflammation. In fact, most inflammatory mediators that affect the growth of cancer act during the stage of promotion. Moreover, inflammation-inducing factors are also frequently cancer promoters [38]. The mechanisms used by inflammatory condition to promote tumor growth are numerous and extremely complex. Apart from enhanced proliferation or increased survival they may also involve the activation of angiogenesis and metastasizing. This is associated, among others, with the production by the transforming cancer cells of many cytokines and chemokines which attract various populations of immune cells, including macrophages, neutrophils, adipose cells, dendritic cells and T, B and NK cells, which in turn infiltrate tumor tissues and form tumor microenvironment [39]. Leukocytes may account for as many as 50 % of total tumor mass. The interaction between cancer cells and macrophages stimulates them to produce such cytokines as IL-8, which stimulates further inflow of inflammatory cells. In tumor microenvironment, cells communicate with one another either by direct contact or production of various mediators [40].

Concluding, inflammatory processes can modulate the course of cancer, inhibiting or stimulating its growth. The activity of inflammatory cells, and the type and level of the inflammation-modulating factors affect the balance between their pro- and antitumor effects. In the initial phase of cancer growth, the acute phase responses may show anticancer action, as it should be remembered that the acute inflammatory reaction in itself is a physiological reaction developing in response to a damaging stimulus and aimed to eliminate the stimulus. It has been shown that in certain types of cancers the ability to induce controlled acute inflammatory condition is used as a therapy component. However, in a chronic inflammatory state, especially in tumor microenvironment, the presence of inflammatory cells acts rather for the benefit of cancer cells, stimulating their survival and proliferation.

The thorough knowledge and understanding of the mechanisms that associate inflammation with neoplastic disease may provide tangible benefits both in the scientific and clinical aspects related to the introduction of new diagnostic and therapeutic methods. The assessment of the involvement of inflammation in respective neoplastic lesions may affect the preventive and therapeutic measures in patients with chronic inflammations.
